# Sentinel Lymph Node Mapping in Presumed Low- and Intermediate-Risk Endometrial Cancer Management (SLIM): A Multicenter, Prospective Cohort Study in The Netherlands

**DOI:** 10.3390/cancers15010271

**Published:** 2022-12-30

**Authors:** Lara C. Burg, Roy F. P. M. Kruitwagen, Annemarie de Jong, Johan Bulten, Tijmen J. J. Bonestroo, Arjan A. Kraayenbrink, Dorry Boll, Sandrina Lambrechts, Huberdina P. M. Smedts, Annechien Bouman, Mirjam J. A. Engelen, Jenneke C. Kasius, Ruud L. M. Bekkers, Petra L. M. Zusterzeel

**Affiliations:** 1 Department of Gynaecological Oncology, Radboud University Medical Center, 6500 HB Nijmegen, The Netherlands; 2 Department of Obstetrics and Gynaecology, Maastricht University Medical Center, 6202 AZ Maastricht, The Netherlands; 3 GROW—School for Oncology and Reproduction, Maastricht University, 6200 MD Maastricht, The Netherlands; 4 Department of Pathology, Radboud University Medical Center, 6500 HB Nijmegen, The Netherlands; 5 Department of Obstetrics and Gynaecology, Rijnstate Hospital, 6800 TA Arnhem, The Netherlands; 6 Department of Obstetrics and Gynaecology, Catharina Hospital, 5602 ZA Eindhoven, The Netherlands; 7 Department of Obstetrics and Gynaecology, Amphia Hospital, 4800 RK Breda, The Netherlands; 8 Department of Obstetrics and Gynaecology, Deventer Hospital, 7400 GC Deventer, The Netherlands; 9 Department of Obstetrics and Gynaecology, Zuyderland Medical Center, 6130 MB Heerlen and Sittard-Geleen, The Netherlands; 10 Department of Gynecological Oncology, Amsterdam University Medical Centres, Centre for Gynecological Oncology Amsterdam (CGOA), 1100 DD Amsterdam, The Netherlands

**Keywords:** endometrial cancer, low-risk, intermediate-risk, sentinel lymph node mapping, indocyanine green

## Abstract

**Simple Summary:**

Sentinel lymph node (SLN) mapping is safe, feasible, and cost-effective to determine the lymph node status in endometrial cancer (EC). The aim of our prospective SLIM study was to investigate the incidence of SLN metastases and the contribution of SLN mapping in the management of presumed low- and intermediate-risk EC, i.e., clinical early-stage EC, endometrioid histology, grade 1 or 2. SLN metastases were present in 11.2% of 152 patients. Adjuvant management was adjusted based on the SLN status in 7.9% of patients: in 5.9% adjuvant treatment was added due to a positive sentinel node, and in 2.0% adjuvant treatment was limited due to unexpected grade 3 disease with a negative SLN. SLN mapping seems important in patients with presumed low- and intermediate-risk EC and may avoid undertreatment as well as overtreatment.

**Abstract:**

The aim was to investigate the incidence of sentinel lymph node (SLN) metastases and the contribution of SLN mapping in presumed low- and intermediate-risk endometrial cancer (EC). A multicenter, prospective cohort study in presumed low- and intermediate-risk EC patients was performed. Patients underwent SLN mapping using cervical injections of indocyanine green and a minimally invasive hysterectomy with bilateral salpingo-oophorectomy. The primary outcome was the incidence of SLN metastases, leading to adjusted adjuvant treatment. Secondary outcomes were the SLN detection rate and the occurrence of complications. Descriptive statistics and univariate general linear model analyses were used. A total of 152 patients were enrolled, with overall and bilateral SLN detection rates of 91% and 61%, respectively. At final histology, 78.9% of patients (*n* = 120) had truly low- and intermediate-risk EC. Macro- and micro-metastases were present in 11.2% (*n* = 17/152), and three patients had isolated tumor cells (2.0%). Nine patients (5.9%) had addition of adjuvant radiotherapy based on SLN metastases only. In 2.0% of patients with high-risk disease, adjuvant therapy was more limited due to negative SLNs. This study emphasizes the importance of SLN mapping in presumed early-stage, grade 1 and 2 EC, leading to individualized adjuvant management, resulting in less undertreatment and overtreatment.

## 1. Introduction

The incidence of endometrial cancer (EC) in the western world is increasing, due to the increasing prevalence of obesity, a prolonged life expectancy, and changes in reproductive behavior [1]. The majority of patients (80%) are defined as presumed early-stage, with the tumor clinically and/or radiologically confined to the uterus, and grade 1 or 2 endometrioid histology on endometrial sampling. Defining the optimal treatment strategy for this patient group is of utmost importance. In contrary to most other countries, the standard surgical procedure in presumed low- and intermediate-risk EC in the Netherlands consists only of a total hysterectomy and bilateral salpingo-oophorectomy, without taking the lymph node status into account [1]. Preoperative myometrial invasion, nor biomarkers, are implemented in the preoperative risk classification. These patients are not referred to an oncology unit, unlike in all other gynaecological malignancies. Adjuvant treatment is based on postoperative patient and tumor characteristics, i.e., age, tumor stage, tumor histology and grade, depth of myometrial invasion, and the presence of lymphovascular space invasion (LVSI). The prognosis of early-stage low- and intermediate-risk EC is generally reassuring with overall survival rates of 80% to 90%, but in approximately 10% of patients with presumed early-stage EC lymph node metastases occur. Consequently, those patients are undertreated, resulting in a significant decreased disease-free and overall survival of 50–60% [2,3,4,5,6].

Worldwide, there is still no clear consensus on the surgical management of presumed early-stage, low- and intermediate-risk EC [7,8,9]. Internationally, there is ongoing debate about whether or not to perform a lymphadenectomy [10,11]. The ASTEC trial and the trial by Benedetti Panici et al. found no evidence for a therapeutic effect of a routine lymphadenectomy itself, and a lymphadenectomy does increase the risk of surgery-related morbidity and lymphedema [12,13,14,15]. These studies did not examine if adapting adjuvant therapy in case of a lymph node metastasis leads to improved survival. If the lymph node status is not examined, there is a risk of understaging, and subsequently the determination of adjuvant therapy is only based on a risk assessment. This may result in overtreatment of patients with a high-risk profile without lymph node metastases, and undertreatment of patients with a low-risk profile with lymph node metastases [16,17,18]. Sentinel lymph node (SLN) mapping has increasingly emerged as a method to determine the lymph node status with a relatively limited extent of surgery with very little morbidity and a high accuracy [19,20,21,22]. It is performed with a cervical injection of a tracer at the start of the surgical procedure. The use of indocyanine green (ICG) as tracer results in higher detection rates and higher negative predictive values compared to the traditional dyes. Overall SLN detection rates of 92% to 97% and negative predictive values of 96% to 99% have been reported [7,20,23,24]. As the involvement of lymph nodes is one of the most important prognostic factors, adding SLN mapping in presumed early-stage grade 1 or 2 EC patients may further improve outcomes for these patients.

The primary objective of this multicenter, prospective, cohort study was to examine the incidence of SLN metastases and the contribution of SLN mapping in the management of patients with presumed low- and intermediate-risk EC. Secondary objectives were to investigate the detection rate of SLN mapping and the risks and complications of SLN mapping.

## 2. Materials and Methods

### 2.1. Study Design

The SLIM study was a multicenter, prospective, cohort study in women with presumed low- and intermediate-risk EC. Patients were enrolled from eight different hospitals: four tertiary referral hospitals and four general teaching hospitals. In each of the participating hospitals, a minimum number of thirty EC patients had to be treated surgically each year. Another requirement was that each participating gynaecologist had performed at least fifty laparoscopic hysterectomies. Before the start of participation of each of the participating hospitals, the principal investigator (PZ) performed a physical or digital site visit to instruct the surgeons and to confirm standardisation of the procedures. A proctoring gynaecologist-oncologist was available in the first number of cases (varying between three and ten cases), as for most general gynaecologists SLN mapping was a relatively new procedure.

Patients were eligible to participate in the study if they (A) were 18 years or older, (B) had preoperative histologically confirmed endometrioid EC grade 1 or 2, (C) had a presumed low- or intermediate-risk profile [25], and (D) had a normal serum CA-125, or an increased serum CA-125 but no evidence of metastases on CT-scan. Classification of low- and intermediate-risk EC was performed according to the ESGO/ESTRO/ESP guidelines for the management of patients with endometrial carcinoma, without the aid of molecular characterizations [25]. The degree of myometrial invasion was no inclusion criterion, as this cannot be assessed completely reliable in the preoperative phase. Patients were excluded from participation if they had: an intermediate-risk profile based on a grade 3 tumor, a high-risk profile, a contraindication to minimal invasive surgery, or anaesthesia, previous pelvic or abdominal radiation, a previous allergic reaction to indocyanine green or iodine, a previous pelvic or para-aortic lymph node dissection or sampling for a previous malignancy, evidence of pelvic and/or distant metastasis, pregnancy, severe renal impairment, or hyperthyroidism. Patient eligibility was reviewed per hospital. Ethical approval was obtained by the Radboudumc Committee for Ethics in Research (‘CMO’) in the region Arnhem and Nijmegen (file number 2015-1783) and Institutional Review Board approval at each site. Written informed consent was obtained from all eligible patients.

To calculate the required number of patients, a sample size calculation was performed. The null-hypothesis (H0) was that the addition of SLN mapping to the standard Dutch care (i.e., hysterectomy and bilateral salpingo-oophorectomy) will result in 10% treatment change in presumed early-stage grade 1 and 2 EC [26]. This design yielded a type I error of 0.05 and power of 0.8. The sample size was calculated by using the following data: a prevalence of lymph node metastases in presumed low- and intermediate-risk EC of 8–12% [27,28,29,30,31,32], a distribution in preoperative histological grade 1 and 2 EC (by biopsy or (micro-) curettage) of, respectively, 75% and 25%, an agreement in pre- and postoperative histological grading of 95% [33], a unilateral detection rate of SLN mapping of 90%, and a bilateral detection rate of 70% [24]. With 10% presumed lost to follow-up, this resulted in a sample size of 152 patients.

SLN mapping was performed using the cervical injection technique with ICG: after the introduction of anesthesia, ICG (1.25 mg/mL) was injected into the cervical submucosa and stroma through a spinal needle at 3 and 9 o’clock positions, superficial (1–2 mm) and deep (20–30 mm), 1 mL per injection with a total of 4 mL. The SLNs were localized by the use of a near-infrared fluorescence camera (Firefly™ Fluorescence Imaging for da Vinci^®^ Xi ™; D-Light P system for Storz; near-infrared system for Olympus). SLNs were identified as currently described in the ‘Operation Guide’ by Moloney et al.: preserving and dividing of the Round and Infundibulopelvic ligament, identification of the external iliac vessels, internal iliac artery, ureter, umbilical ligament, and uterine artery, the opening of the paravesical space, pursuing the lymphatic channels up to the lymph node, and dissection of the lymph node in the pelvic or, rarely, para-aortic region [34]. If multiple lymphatic pathways were seen then both draining lymph nodes were dissected. Frozen section of SLN specimens was not performed. Subsequently, a laparoscopic or robot-assisted hysterectomy with bilateral salpingo-oophorectomy was performed. In case SLN mapping failed one- or both-sided, only a hysterectomy with bilateral salpingo-oophorectomy was performed, in correspondence with the current Dutch guidelines for presumed early-stage EC [1].

Consecutive patients were enrolled in the SLIM study from the start of SLN mapping in the participating hospitals. The principle was followed that if ICG was injected and the retroperitoneal space was opened, patients were included in the analysis, regardless of SLN mapping result. Patients who withdrew before the study intervention or who did not receive the study intervention (injection of ICG and opening of the retroperitoneal space) were not included in the analyses.

All removed SLNs, as well as the uterus, Fallopian tubes, ovaries, and—if present—other removed lymph nodes were analysed by specialized gynaecological pathologists. The participating pathology laboratories used a protocol in which the SLN was serially sectioned. Each SLN was sliced perpendicular to the longest axis in 2–3 mm slices. The slices were routinely processed after formalin fixation. The paraffin block was cut until the contour of the lymph node appears. Then, 4 µm sections were cut into 6 ribbons with step series spaced 200 µm apart. A central section of ribbon 4, 5 and 6 was stained with hematoxylin and eosin and an adjacent section for keratin immunohistochemical staining using the monoclonal antibody Cytokeratin 8.18 (Cam5.2; BD, OMNIS retrieval 1:20). SLN metastases were defined according to the common definitions of macro-metastasis (>2 mm), micro-metastasis (0.2–2 mm), and isolated tumor cells (ITC; <0.2 mm). Non-sentinel lymph nodes were handled according to institutional standard-of-care practices, and therefore negative H&E slides from non-sentinel lymph nodes were not subjected to immunohistochemistry. 

Participating surgeons provided encrypted data of all included patients on patient characteristics, surgical procedure, pathological assessment, and peri- and postoperative complications and adverse events. Determination of the oestrogen receptor (ER) and progesterone receptor (PR) status was not routinely performed, but added to the pathological assessment in case of a positive SLN.

Adjuvant management was based on the current Dutch guidelines, local protocols, and the presence or absence of lymph node metastases [1]. The presence of a macro- or micro-metastasis resulted in FIGO stage IIIC disease; those patients received adjuvant external beam radiotherapy (EBRT) and/or chemotherapy. Patients with only ITC were managed with a watchful waiting policy, without specific adjuvant therapy. The observation was performed through CT scans of the pelvis and abdomen six and twelve months after surgery.

Patients with postoperative FIGO stage IA, low- and intermediate-risk EC, were not treated with adjuvant therapy. Patients with postoperative FIGO stage IB, low- and intermediate-risk EC, did not receive adjuvant therapy or were treated with vaginal brachytherapy when older than 60 years [35]. Patients with FIGO stage II, low- and intermediate-risk EC were treated with external beam radiotherapy, depending on histological findings (irradicality, myometrial invasion, and LVSI). The absence of SLN metastases in patients with FIGO stage II EC led in some cases to the advice for a smaller field of external beam radiation, following local protocol. Patients with FIGO stage I-II EC could be included in the PORTEC 4A study if they met the inclusion criteria of that study. Those patients were randomized in the experimental group (molecular-integrated risk profile-based adjuvant treatment) or the standard group (adjuvant vaginal brachytherapy) [36]. 

Patients with of presumed low- and intermediate-risk EC, but who postoperatively turned out to have high-risk features, had an indication for determining the lymph node status. In case of successful SLN mapping, an extra operative procedure could be omitted.

### 2.2. Statistical Analyses

Descriptive statistics were used to describe the study population, overall and bilateral SLN detection rate, prevalence of SLN metastases, risk factors for SLN metastasis, the contribution of SLN mapping to the adjustment of adjuvant treatment, and complication rate. Univariate general linear model analyses were used to study relations between determinants (e.g., age, histological grade, tumor size, etc.) and the main outcome (SLN metastasis). A *p*-value less than 0.05 was considered statistically significant. All analyses were performed with SPSS for Windows Version 25, release 25.0.0.1.

## 3. Results

**Figure 1:**
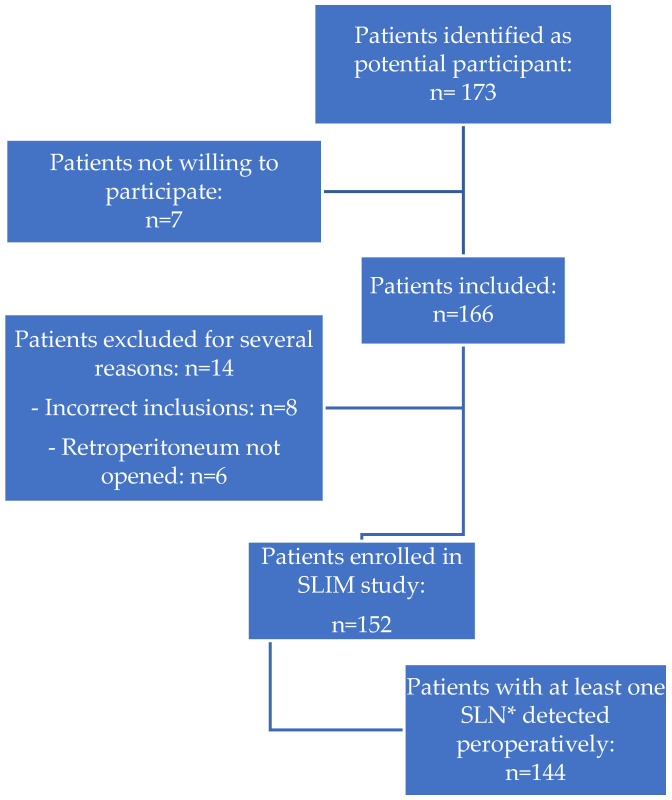
Identification and inclusion of patients in the SLIM study. * SLN = sentinel lymph node.

Between March 2016 and December 2021, 178 patients were identified as potential participants for the SLIM study. Twelve patients were not willing to participate, resulting in 166 inclusions. Of them, fourteen patients were excluded due to an incorrect inclusion (*n* = 8, reasons included poor general condition of a patient, patients’ withdrawal, or patients who met one of the exclusion criteria but signed the informed consent form), or because the retroperitoneum had not been opened during surgery (*n* = 6, reasons included anaesthetic difficulties during surgery, conversion to laparotomy, lack of time due to COVID-19 restrictions in surgery time). In the end, 152 patients with presumed early-stage, grade 1 or 2 EC (considered as presumed low- and intermediate-risk EC) were enrolled and underwent SLN mapping with ICG, followed by hysterectomy and bilateral salpingo-oophorectomy (Figure 1). Most of these patients were postmenopausal (88%), with a median age of 66 years (range 39–84 years). The median body-mass index of patients was 29.7 kg/m^2^ (range 17.6–52.2 kg/m^2^); 49% was obese (BMI > 30 kg/m^2^). Clinical, surgical, and pathological characteristics are shown in Table 1. The preoperative pathological assessment showed a grade 1 endometrioid tumor in 74% of patients and a grade 2 endometrioid tumor in 26% of patients; final histology, however, showed grade 3 or non-endometrioid EC in 8% of patients.

### 3.1. Detection Rate of SLN Mapping

A total of 264 SLNs were dissected in 152 patients. The majority of SLNs were found in the external iliac region (38%), the obturator loge (34%), and the internal iliac region (17%). In 9% of patients, the SLN was found outside the standard region for pelvic lymph node dissection (i.e., pre-sacral, para-aortal, para-caval). In 95% of the procedures, at least one SLN was detected. Bilateral surgical SLN detection was successful in 76%. In patients with successful SLN mapping, the mean number of removed SLNs per hemipelvis was 1.2 lymph nodes. Postoperative pathological assessment of the assumed lymph node tissue showed an empty packet dissection (a dissection that does not yield a lymph node on pathological assessment) in nineteen patients (13%): in fourteen patients with assumed bilateral SLN mapping only a unilateral SLN was dissected, in two patients with assumed bilateral mapping no SLN was dissected at all, and in three patients with assumed unilateral mapping no SLN was dissected. This led to pathological SLN detection rates of 91% and 61% for overall and bilateral mapping, respectively.

### 3.2. Incidence of SLN Metastases

Macro- and micrometastases were found in seventeen of 152 patients (11.2%). Three patients (2.0%) had ITC. Lymphovascular space invasion (LVSI) was present in eleven of these 20 patients with a positive SLN (55%).

Eleven of the seventeen patients with macro- and micrometastases (64.7%), had grade 1–2 endometrioid EC. In final histology, nine of the eleven patients with grade 1 or 2 EC with a metastasized sentinel node, were LVSI positive. Pre-operatively, LVSI status was available in the minority (33.8%) of the included patients. Table 2 shows the characteristics of patients with and without SLN metastases (excluding the nineteen patients without successful (i.e., at least unilateral) SLN mapping, or with an empty packet dissection).

Univariate analyses showed age >60 years (*p* < 0.05), postoperative histological grade (*p* < 0.001), myometrial invasion (*p* < 0.005), and LVSI (*p* < 0.001) to be statistically significant predictors for SLN metastasis. The preoperative histological grade in itself, without other biomarkers, was no significant predictor. In patients with SLN metastases, ER and PR status were positive in 70% and in 20% only ER was positive and PR negative. In just one patient with SLN metastases, ER and PR were both negative.

Of all 152 included apparent early-stage grade 1 or 2 EC patients (apparent low- and intermediate-risk EC) only 78.9% (*n* = 120) were truly low- and intermediate-risk. The other 32 patients were high-risk post-operatively due to multiple reasons: seven patients had grade 3 EC (of whom four had SLN metastases), five patients had non-endometrioid EC (of whom two had SLN metastases), twelve patients with grade 1–2 EC had SLN metastases, two patients with grade 1–2 EC were postoperatively upstaged due to extension beyond the uterus with SLN metastases, and six patients with grade 1–2 EC were postoperatively upstaged due to extension beyond the uterus without SLN metastases. In thirteen of these 120 patients with true low- or intermediate-risk EC (10.8%) LVSI was present. If only the presence of LVSI was considered as an independent risk-factor that changes the risk-classification into high-risk EC, only 70.4% of the patients with apparent low- or intermediate-risk EC were correctly categorized correctly pre-operatively.

### 3.3. Adjusting Adjuvant Management Based on Lymph Node Status

Four of the seventeen patients with macro- and micrometastases also had another indication for adjusting the adjuvant management independent of their lymph node metastases, due to ovarian metastases (*n* = 1), non-endometrioid postoperative histology (*n* = 2), or FIGO stage II disease (*n* = 1). 

In the remaining thirteen patients with macro- or micrometastases, adjuvant management was adjusted purely based on SLN mapping. Nine of them (5.9% of all patients) had a grade 1 or 2 endometrioid tumor after final pathological assessment without other extension beyond the uterus. SLN mapping in these patients was the only indication for upstaging to FIGO stage III disease and for adjuvant therapy: three patients were treated with external beam radiotherapy followed by chemotherapy and six patients received adjuvant external beam radiotherapy alone. The other four patients had SLN metastases but postoperative grade 3 EC. Those patients received adjuvant chemotherapy, with or without external beam radiotherapy.

In the three patients with ITC (all with grade 1 or 2 endometrioid EC), no adjustment in adjuvant therapy was made, but a watchful waiting policy was performed. Two patients underwent a CT scan after six and twelve months, showing no abnormalities. The third patient underwent only one CT scan eight months after surgery, also showing no abnormalities.

132 patients had no metastasized SLN. However, no SLN was detected in thirteen of them, and in 36 patients the SLN was found only unilaterally, leaving 83 patients with a negative SLN bilaterally. LVSI was present in 13% of patients with a negative SLN (*n* = 15). In patients with grade 3 EC and successful bilateral SLN mapping, no staging surgery was needed after final histology as lymph node status was already known. Twenty patients without SLN metastases had an indication for vaginal brachytherapy. Six patients had FIGO stage II EC, and were treated with adjuvant external beam radiotherapy. In three of them, a smaller field of external beam radiation was given following local protocols (only on the uterus region), due to the negative lymph nodes.

**Figure 2:**
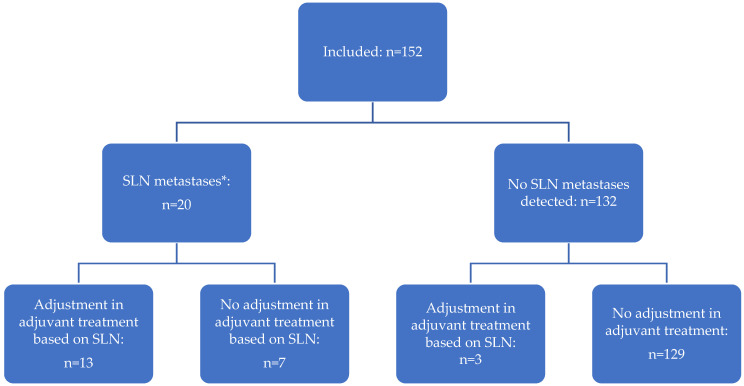
The contribution of SLN mapping in the management of patients with presumed early-stage, low- and intermediate-risk EC. * Includes macro-metastases (*n* = 10), micro-metastasis (*n* = 7), and isolated tumor cells (*n* = 3).

In total, adjuvant treatment was adjusted based on performing SLN mapping in twelve of 152 patients (7.9%): in nine patients (5.9%) undertreatment was prevented as they received adjuvant treatment due to SLN mapping, whereas in three patients (2.0%) with grade 3 EC in final histology overtreatment was prevented by treating them with a smaller irradiation field that did not include the pelvic node region (Figure 2).

### 3.4. Complications of SLN Mapping

A total of nine complications during surgery (5.9%) and nineteen complications in the postoperative phase (13%) were registered (Table 1). Only one surgical complication could directly be linked to SLN mapping, i.e., obturator nerve injury. One postoperative complication was linked to SLN mapping, i.e., lymphedema, and one complication may be linked to SLN mapping (i.e., a numbness feeling in the groins). All other complications were due to the hysterectomy.

## 4. Discussion

The SLIM study examined the contribution of SLN mapping by using ICG in the management of patients with presumed low- and intermediate-risk EC. The results of the SLIM study show that SLN mapping leads to less undertreatment of patients with presumed low- and intermediate-risk EC (presumed stage I grade 1 or 2 EC), due to upstaging if SLN metastases are present. Overtreatment can be prevented as well, due to limited therapy when final histology shows grade 3 EC without SLN metastases.

SLN metastases were found in up to 11.2% of patients with presumed pre-operative low- and intermediate-risk EC, and another 2.0% had ITC. Previous studies show comparable percentages of SLN metastases [2,3]. In the SLIM study, we calculated the percentage of lymph node metastases based on the included 152 patients. As SLN mapping had a bilateral surgical detection rate of only 76% (61% pathological), this percentage may therefore be an underestimate.

Only 78.9% of the included presumed low- and intermediate-risk EC patients were truly low- and intermediate-risk in the postoperative assessment, as the others had grade 1 and 2 EC with SLN metastases, or high-risk EC with or without SLN metastases. LVSI was present in 10.8% of the patients with true low- and intermediate-risk EC. Although these patients remained included in the low- and intermediate-risk group, as in the Netherlands LVSI is not yet implemented in the risk classification and the presence of LVSI in itself does also not lead to an adjustment in adjuvant therapy in the Netherlands, we showed that LVSI was an important risk-factor for lymph node metastases.

The preoperative identification of true low- and intermediate-risk patients versus high-risk patients remains difficult [33]. We showed that pre-operative grade 1 or 2 histology is not sufficient in predicting whom is at risk for lymph node metastases. Pre-operative characteristics, specific ultrasounds, other imaging techniques, and biomarkers, whether or not implemented in a risk model, may improve pre-operative screening to identify whom is at risk for lymph node metastases. Further studies are needed to assess if risk models may adequately identify a subgroup of patients in whom SLN detection is futile, especially since SLN mapping is cost-effective and associated with a low complication rate [37]. Furthermore, patients are willing to choose for SLN mapping, if the risk of lymph node metastases is small [38].

This study showed that SLN mapping does reduce undertreatment in patients with SLN metastases, and also reduces overtreatment. Three patients with FIGO stage II EC were treated with a smaller irradiation field than usual due to a negative SLN. In the Netherlands, this is not common practice, but per case decided in multidisciplinary meetings and conform local protocols. An advantage of this policy is that more limited radiotherapy leads to fewer side effects. Furthermore, it is supposed to be safe to limit the irradiation field, as literature exists stating that even vaginal brachytherapy alone might be sufficient to avoid recurrence for these patients, without a statistically significant difference in overall survival [39]. Another group of patients in which overtreatment might be reduced due to SLN mapping, is the group of presumed low- and intermediate-risk EC patients, who postoperatively turned out to have high-risk features (non-endometrioid EC, or grade 3 endometrioid EC, or extension beyond the uterus). A second surgery to perform a lymphadenectomy can be omitted, as the lymph node status is already known due to SLN mapping. The high diagnostic accuracy of SLN mapping is confirmed by other studies in both low- and intermediate-risk and high-risk EC [20,40,41,42,43,44,45], and SLN mapping is considered to be at least evenly safe as a (pelvic) lymphadenectomy, with a higher detection rate of SLN metastases compared to a lymphadenectomy [7]. This is probably due to the identification of lymph nodes located outside the routine lymphadenectomy area (17% of SLN metastases in the FIRES trial were found in non-traditional sites [42]), and the use of ultrastaging. Therefore, patients with postoperative high-risk features and successful SLN mapping do not have to be scheduled for a subsequent staging procedure [46,47].

We showed that SLN mapping is a feasible technique, with an overall (bilateral and/or unilateral) surgical detection rate of 95% and a bilateral surgical detection rate of 76% when using ICG as the tracer. Although multiple tracers and injection localisations can be used, current literature favours the use of a cervical injection with ICG because of the highest SLN detection rates [19,20]. Nevertheless, empty packet dissection is common during the learning curve of SLN mapping, especially in patients with a higher BMI or age [44]. Previous studies showed that the learning curve of SLN mapping in EC is achieved after thirty to forty cases, and that the risk of empty packet dissection decreases with the number of performed mappings, in which each additional procedure leads to a 3.6% reduction in the odds of an empty packet [48,49,50]. In the SLIM study, the majority of empty packet dissections were found within the first twenty consecutive cases.

As shown in other studies, we confirmed in our study that SLN mapping is a safe procedure with low complication rates [40,43,51]. This is important, since we assess SLN mapping in a low- and intermediate-risk population. A dilemma that comes with SLN mapping is the presence of ITC. The presence of macro- and micro-metastases has therapeutic consequences since previous studies show a beneficial effect of adjuvant chemotherapy compared to radiotherapy alone in FIGO stage IIIC EC [16,17]. However, detailed pathological assessment with ultrastaging (i.e., serial sectioning and immunohistochemistry) might lead to the identification of isolated tumor cells <0.2 mm. In the SLIM study, patients with ITC did not receive adjuvant therapy (unless tailored to uterine factors). In other malignancies such as breast cancer, gastrointestinal cancers, and melanoma, ITC are related to an increased risk of recurrence and worsened prognosis [52,53,54]. In EC, there is still a lot of discussion on this topic worldwide. A prospective single centre study reported that patients with ITC have a good prognosis and that adjuvant therapy should be based on uterine factors instead of the presence of ITC [23]. This opinion is shared by other research groups [55], even though there are also groups who are more careful and suggest that the chance of recurrent disease is higher without adjuvant therapy [56]. In a web-based survey, sent by email to all members (gynaecologic oncologists) of the European Society of Gynaecological Oncologists, International Gynaecological Cancer Society, and Society of Gynaecological Oncologists, 14% of respondents recommended adjuvant therapy if ITC were identified [57].

In countries where a pelvic lymphadenectomy is a common strategy in low- and intermediate-risk EC, it might be straightforward to change the policy to SLN mapping. However, SLN mapping in presumed low- and intermediate-risk EC remains controversial in countries that use the risk stratification (low-, intermediate-, high-risk) for the indication for adjuvant therapy, such as the Netherlands and the United Kingdom. Arguments for performing SLN mapping are the number of SLN metastases in low- and intermediate-risk EC, the widespread acceptance of SLN mapping in other countries (more than 50% of international gynaecological oncologists adopted SLN mapping [57]), and the implementation of SLN mapping in international guidelines [7]. However, multiple dilemmas should be appointed. First, although SLN mapping likely leads to better oncological safety, high-level evidence on overall and disease-free survival benefit is lacking [14,15,22,51,58,59]. The PORTEC-3 trial showed beneficial effects of adjuvant chemotherapy on recurrence-free survival in FIGO stage III disease, but more research is necessary to examine the impact of SLN mapping on long-term oncological outcomes [16]. Other study groups are currently prospectively investigating the oncological safety of SLN mapping, however they do not specifically focus on presumed low- and intermediate-risk EC [60,61]. Their results are expected in the upcoming years. We, therefore, intend to continue to follow our SLIM study population, and to expand the population, to gain more knowledge on long-term oncological outcomes. Second, implementation of SLN mapping in a country such as the Netherlands might be more complicated since the presumed low- and intermediate-risk EC patients are mostly treated in the general hospitals by general gynecologists, and not by a gynaecological oncologist in one of the tertiary (academic) hospitals [1,62].

If no SLN was found during surgery, no pelvic and/or para-aortic lymphadenectomy was performed in the patients included in the SLIM study. This is because we adhered to the current Dutch guidelines, which do not include any kind of lymph node sampling in low- and intermediate-risk EC, in contrary to the NCCN SLN algorithm [8]. Our previous cost-effectiveness analysis showed that SLN mapping is cost-effective compared to the risk stratification strategy as used in the Netherlands (adjuvant therapy based on patient and uterine risk factors) and a lymphadenectomy [37]. With the obesity pandemic and the relationship between EC and obesity, this might become even more important. In case of unilateral SLN mapping, it is cost-effective to base adjuvant treatment on patient and uterine risk factors [37]. Unilateral SLN mapping will, however, become less common after performing multiple procedures and achieving the aforementioned learning curve [48,49]. Presumed low- and intermediate-risk EC patients, therefore, do not necessarily need to be treated by gynaecological oncologists. Furthermore, our previous study on patients’ and gynecologists’ views on SLN mapping showed that patients are willing to travel to another hospital, and that gynecologists on the other hand are willing to refer their patients to another hospital if SLN mapping is offered there [38].

This study also has some points that need special consideration. First, the design of our study was a prospective cohort study. Consultation of patient focus groups taught us that patients were not willing to be randomized between SLN mapping versus no lymph node dissection, as patients gained knowledge of the stage of the disease with only a limited addition to the standard surgical treatment; furthermore, SLN mapping is already implemented in international guidelines. Second, the inclusion of patients started slowly, since the approval by the ethical committees of the participating hospitals took longer than expected and a proctoring gynaecological oncologist had to be available for the first cases in the majority of the participating hospitals. This resulted in a rather long inclusion period (2016–2021). The majority of patients (75%) were included between 2019 and 2022.

## 5. Conclusions

This study showed that in 11.2% of patients with presumed low- and intermediate-risk EC lymph node metastases are present, and in 5.9%, adjuvant therapy was adjusted purely based on SLN metastases. As preoperative selection of true low- and intermediate-risk EC patients remains difficult, with only 78.9% of the included patients truly having a low- and intermediate-risk profile, we recommend considering SLN mapping in the management of presumed low- and intermediate-risk EC.

## Figures and Tables

**Table 1:** Clinical, surgical and pathological characteristics.

Characteristics	Patients (*n* = 152)
Age in years, median (range)	66 (39–84)
Menopausal status	
Premenopausal (%)	19 (12)
Postmenopausal (%)	133 (88)
BMI in kg/m^2^, median (range)	29.7 (17.6–52.2)
<25 kg/m^2^ (%)	30 (20)
>25 kg/m^2^ (%)	122 (80)
Preoperative histological grade, *n* (%)	
Grade 1	113 (74)
Grade 2	39 (26)
Postoperative histological grade, *n* (%)	
Grade 1	107 (70)
Grade 2	33 (22)
Grade 3	5 (3)
Non-endometrioid	7 (5)
Time in minutes between ICG ^1^ injection and removal of SLNs, mean (range)	27 (5–90)
Number of removed SLNs ^2^ per hemipelvis, mean	1.2
Peroperative (surgical) SLN detection, *n* (%)	
No detection	8 (5)
At least unilateral detection	144 (95)
Bilateral detection	116 (76)
Postoperative (pathological) SLN detection, *n* (%)	
No detection	13 (9)
At least unilateral detection	139 (91)
Bilateral detection	100 (61)
SLN metastasis, *n* (%)	
No SLN detection	13 (8.6)
None	119 (78)
Macro-metastasis	10 (6.6)
Micro-metastasis	7 (4.6)
Isolated tumor cells	3 (2.0)
Postoperative FIGO stage, *n* (%)	
IA	84 (55)
IB	39 (26)
II	6 (4)
IIIA	6 (4)
IIIB	0
IIIC	17 (11)
IV	0
Complications	
Peroperative	9 (6.0) ^3^
Postoperative	19 (13) ^4^

^1^ ICG = Indocyanine Green. ^2^ SLN = sentinel lymph node. ^3^ One complication is linked to SLN mapping (0.7%). ^4^ Two complications are possibly linked to SLN mapping (1.3%).

**Table 2:** Characteristics of the patients with andomized SLN mapping (*n* = 139), with and without Sentinel Lymph Node (SLN) metastasis.

Characteristics	Sentinel Lymph Node Positive ^1^ (*n* = 20)	Sentinel Lymph Node Negative (*n* = 119)	*p*-Value
Age			0.041
<60 years	3 (15%)	46 (39%)	
≥60 years	17 (85%)	73 (61%)	
BMI			0.619
<25	5 (25%)	25 (21%)	
≥25	15 (75%)	94 (79%)	
Pre-operative CA-125			0.346
<35 U/mL	14 (70%)	94 (79%)	
≥35 U/mL	6 (30%)	19 (16%)	
Unknown	0	6 (5%)	
Pre-operative histological grade			0.595
Grade 1	14 (70%)	90 (76%)	
Grade 2	6 (30%)	29 (24%)	
Postoperative histological grade			<0.001
Grade 1	9 (45%)	95 (80%)	
Grade 2	5 (25%)	23 (19%)	
Grade 3	4 (20%)	1 (1%)	
Non-endometrioid	2 (10%)	0	
Tumor size			0.566
<20 mm	6 (30%)	34 (29%)	
≥20 mm	12 (60%)	68 (57%)	
Unknown	2 (10%)	17 (14%)	
Myometrial invasion			0.004
None	1 (5%)	26 (22%)	
<50%	5 (25%)	55 (46%)	
≥50%	14 (70%)	38 (32%)	
Lymphovascular space invasion			<0.001
Present	11 (55%)	15 (13%)	
Absent	7 (35%)	102 (86%)	
Unknown	2 (10%)	2 (1%)	
ER/PR status ^2^			n/a
Both positive	14 (70%)	50 (42%)	
Both negative	1 (5%)	0	
ER pos/PR neg	4 (20%)	3 (3%)	
ER/PR unknown	1 (5%)	66 (55%)	

^1^ Includes macro-metastases (*n* = 10), micro-metastasis (*n* = 7) and isolated tumor cells (*n* = 3). ^2^ ER: estrogen receptor, PR: progesterone receptor, NB: a SLN was detected by the pathologist in 139 of 152 patients.

## Data Availability

The data presented in this study are available on request from the corresponding author. The data are not publicly available due to privacy restrictions.
